# Statistics

**Published:** 1880-01

**Authors:** J. B. Chandler


### STATISTICS.

J. B. CHANDLER.

The statistician spends his life in collecting, collating, adding, dividing, subtracting and multiplying ; his business seems to be confined to producing immense tables and their results in figures, and keeping an account of them as they differ from year to year. His life, however, is well spent, and when these results are taken hold of by the political economist, the merchant, or the professional man, and great problems of public utility, mercantile venture, or of law or medicine, are solved thereby, then we come to appreciate the value of the services of the man of figures.

Again, these tables furnish food for thought for those who will think, and oftentimes very profitable thought.

Our attention was arrested a few days since by a paragraph in a newspaper professing to give approximate statistics relative to the population of the world.

The first impression made on the mind by the array of figures was that of the inconsequence of any single individual life to the whole. We felt that we shared with every human being that peculiar isolation of self, the personal individuality which tempts one into the idea that within ourselves lie privileges and exemptions different from other men, gifts which if apparently hidden in higher lights without, still exist enchained in circumstances.

Many go still further and realize the truth of Young's line : "All men think all men mortal but themselves."

And yet our statistician tells us that the world contains just 1,421,000,000 human beings, with physical bodies and mental capacities rivaling our own. We leave to that statistician the labor of collecting the figures and results necessary to solving the problem of human equality at birth, but in our individual case, since the above figures have been placed before us, we feel a sympathy for Daniel Boone, who was crowded because a neighbor squatted within five miles of him. He could move, but the world then did not have fourteen hundred and twenty-one millions of people to make room for.

Now we come to another record, a table to which, sooner or later, each one of us will add a unit, *only a unit.* Think of that, ye princes, potentates and powers ; ye great men of the world. The crowned head, the whining beggar, the successful and the defeated politician, the Empress and the char-woman "to this must come at last;" death makes no distinction, of place, sex nor age; the youngest and the oldest come in alike for Methuselah's record, "and he died."

The tables of mortality show the annual number of deaths to be 35,693,350, or that ninety-seven thousand seven hundred and ninety persons die every day.

This would soon deplete the world of its population were it not for another item, which will probably interest our lady friends.

Let us contemplate those times familiar in almost every household, when the sewing baskets seem overloaded with linen, muslin and laces, when the intrusion of the gentleman of the family is greeted by a hasty hiding of mysterious small unfinished garments; then, the increasing interest, manifested by more frequent calls from lady friends; then, the anxious hours, when desire that all should go well suggests fears that some preparation will be neglected; then, the sacred moments of pain and blessed joy that none but mothers ever realize. We wonder what the heretofore, youngest of the flock will say. Even royalty shares in this, for it is related of the Princess Royal of England, that when she was but a prattling child and was told that she had a little brother, she exclaimed, "How nice, please let me go and tell mamma."

Think of the immense interest manifested at the first tooth, the anxiety about the second summer, the terrors of measles, whooping cough, scarlet fever, diphtheria and "all the ills that *child* flesh is heir to"—think of all these cares concentrated upon our one angel at home, and then consider the tables and find that *in every minute* seventy dear little darling babes are fluttering into this wicked world to contend with its ills, its vices and for its pleasures. Yes, poor anxious mother, when you think yourself blessed above women in your children, or afflicted sorely by their ills or tempers, take the consolation at least that you are not alone, for 104,300 children are born every day, and our statistician does not report a single instance in which there was no mother.

Of course the difference between the mortality and birth lists shows the constant increase of population and the necessity of epidemics, war, famine and other extraneous causes for keeping that increase within bounds.

We leave the thoughts which naturally will arise from a perusal of these figures to each reader to eliminate for himself. They carry a lesson for all and our hope is that some at least will find profit by it.

**Typhoid Fever Propagated.**

In a recent number of the *Popular Science Monthly*, Ely Van De Warker, M. D., of Syracuse, N. Y., under the title "Typhoid Fever Poison," reports seventeen cases of the fever in an isolated suburb of the city in which there were but fourteen houses. The first case was imported; thence through the overflowing of the privy, in which all the excrement of the patient had been thrown, a well became contaminated. All the persons who were taken ill used this well. It was the constant or occasional source of supply of seven of the fourteen families. No cases occurred in the households who did not drink from this well. Some cases were developed in every family who drew water from it. The families who escaped were exposed to every other influence but that of this particular well; their own water supply was the same, less the privy contamination. It is not unlikely that their own wells received some of the overflow from their own vaults, but as these were free from typhoid poison, no ill result ensued.

About eight years since, Dr. Flint, who has studied and written a great deal on the subject, became satisfied that a source of typhoid fever existed which was little dreamed of, and which at first thought would seem impossible. This source, as he then enunciated it to his home medical society (and not to his knowledge having been before suggested), is found in *ice*. If this idea is thoroughly investigated it will not appear to be very problematical. In the first place, the poison is not destroyed or impaired by freezing (some one long ago remarked that ice often masks or conceals what it does not kill). Now, whence comes our ice supply? Often from shallow reservoirs in the midst of neighborhoods of large towns, purposely made to receive surface drainage from all around, under the erroneous idea that no harm will ensue, as freezing is supposed to purify and render harmless what might otherwise be objectionable. Great quantities of ice are taken from canals, from creeks, from stagnant ponds, and from streams that are either the natural or artificial recipients of surface drainage, of the outpourings of sewers, and of uncleanliness from various sources. The danger from ice taken from improper places is not only from that which is drunk, but from its use in refrigerators and preservatories, where milk, butter, fruits, vegetables, and meats are subjected to its saturating influence as it vaporizes. Several instances have fallen under the doctor's observation where the disease, by the most careful investigation, could not be traced to any other source.

